# Cilostazol Attenuates AngII-Induced Cardiac Fibrosis in apoE Deficient Mice

**DOI:** 10.3390/ijms23169065

**Published:** 2022-08-13

**Authors:** Yoshiko Hada, Haruhito A. Uchida, Ryoko Umebayashi, Masashi Yoshida, Jun Wada

**Affiliations:** 1 Department of Nephrology, Rheumatology, Endocrinology and Metabolism, Okayama University Faculty of Medicine, Dentistry and Pharmaceutical Sciences, Okayama 700-8558, Japan; 2 Department of Chronic Kidney Disease and Cardiovascular Disease, Okayama University Faculty of Medicine, Dentistry and Pharmaceutical Sciences, Okayama 700-8558, Japan; 3 Department of Cardiovascular Medicine, Okayama University Faculty of Medicine, Dentistry and Pharmaceutical Sciences, Okayama 700-8558, Japan

**Keywords:** cilostazol, angiotensin II, fibrosis, osteopontin, cAMP-PKA

## Abstract

Cardiac fibrosis is characterized by the net accumulation of extracellular matrix in the myocardium and is an integral component of most pathological cardiac conditions. Cilostazol, a selective inhibitor of phosphodiesterase type III with anti-platelet, anti-mitogenic, and vasodilating properties, is widely used to treat the ischemic symptoms of peripheral vascular disease. Here, we investigated whether cilostazol has a protective effect against Angiotensin II (AngII)-induced cardiac fibrosis. Male *apolipoprotein E*-deficient mice were fed either a normal diet or a diet containing cilostazol (0.1% wt/wt). After 1 week of diet consumption, the mice were infused with saline or AngII (1000 ng kg^−1^ min^−1^) for 28 days. AngII infusion increased heart/body weight ratio (*p* < 0.05), perivascular fibrosis (*p* < 0.05), and interstitial cardiac fibrosis (*p* < 0.0001), but were significantly attenuated by cilostazol treatment (*p* < 0.05, respectively). Cilostazol also reduced AngII-induced increases in fibrotic and inflammatory gene expression (*p* < 0.05, respectively). Furthermore, cilostazol attenuated both protein and mRNA abundance of osteopontin induced by AngII in vivo. In cultured human cardiac myocytes, cilostazol reduced mRNA expression of AngII-induced osteopontin in dose-dependent manner. This reduction was mimicked by forskolin treatment but was cancelled by co-treatment of H-89. Cilostazol attenuates AngII-induced cardiac fibrosis in mice through activation of the cAMP–PKA pathway.

## 1. Introduction

Heart failure (HF) is a major cause of death in the world [1]. Despite progress in the treatment of heart failure in recent decades, prevalence and hospitalization rates are still increasing. Currently, the existing data on HF is estimated to be 26 million adults worldwide, and it is expected to increase continually due to three main factors: aging population, increasing prevalence of comorbidities or risk factors, and improved survival of post-myocardial infarction [2,3]. HF is typically associated with cardiac remodeling. Fibrosis is a fundamental process observed in cardiac remodeling and considered to be a key contributor to heart failure and its progression. Importantly, the presence and extent of myocardial fibrosis has also prognostic implications, as it causes contractile dysfunction and arrhythmias in structural heart disease of various etiologies [4,5,6,7,8,9]. As evidence exists that cardiac inflammation and fibrosis are potentially reversible in experimental and clinical settings, they are important targets for innovative heart failure treatments [10].

Angiotensin II (AngII) plays a critical role in cardiac remodeling [11]. AngII has been relevant to the various cardiovascular diseases, including cardiac hypertrophy and heart failure [12]. While the exact mechanisms by which AngII induces cardiac fibrosis have not been precisely elucidated, recent work supports a critical role for adhesion, a complex process involving interactions between different families of factors, including integrins, extracellular matrix proteins, and adhesion molecules. Osteopontin (OPN), an extracellular matrix protein, is an adhesion protein implicated as one of the important mediators of the profibrotic effects of AngII in heart [11]. Thus, AngII infusion in mice is well established as a murine model of heart failure mainly induced by pressure overload [10].

Cilostazol (6-[4-(1-cyclohexyl-1H-tetrazol-5-yl)butoxy]-3,4-dihydro-2(1H)-quinolinone), a selective inhibitor of phosphodiesterase type III (PDE3) with anti-platelet, anti-mitogenic, and vasodilating properties, is widely used to treat ischemic symptoms of peripheral vascular disease [13,14]. Cilostazol inhibits platelet aggregation by increasing the intracellular cyclic adenosine monophosphate (cAMP). Additionally, cilostazol has demonstrated cAMP pathway-dependent and -independent pharmacological effects, including anti-inflammatory [15,16], antioxidant [17], and anti-apoptotic actions [18,19].

We previously reported that cilostazol attenuated AngII-induced AAA formation by its anti-inflammatory effect through PDE3 inhibition in aortic endothelial cells [20]. Several studies have reported that cilostazol attenuated liver fibrosis [21,22,23]. However, little has been reported the effect of cilostazol on cardiac fibrosis. In this study, we aimed to determine the effects of cilostazol on AngII-induced cardiac fibrosis in apolipoprotein E deficient (apoE^−/−^) mice.

## 2. Results

### 2.1. Cilostazol Had No Effect on Body Weight, Pulse Rate, Systolic Blood Pressure, and Lipid Profile

No significant difference was found in body weight (BW) and pulse rate among the four groups (Table 1). Although AngII infusion significantly increased systolic blood pressure as expected, no difference was observed between the AngII + control diet group and the AngII + cilostazol-containing diet group (Table 1). Accordingly, cilostazol had no effect on total cholesterol, triglyceride, or high-density lipoprotein cholesterol in ApoE^−/−^ mice during saline or AngII infusion (Table 1).

### 2.2. Cilostazol Attenuated AngII-Induced Cardiac Hypertrophy

**Figure 1:**
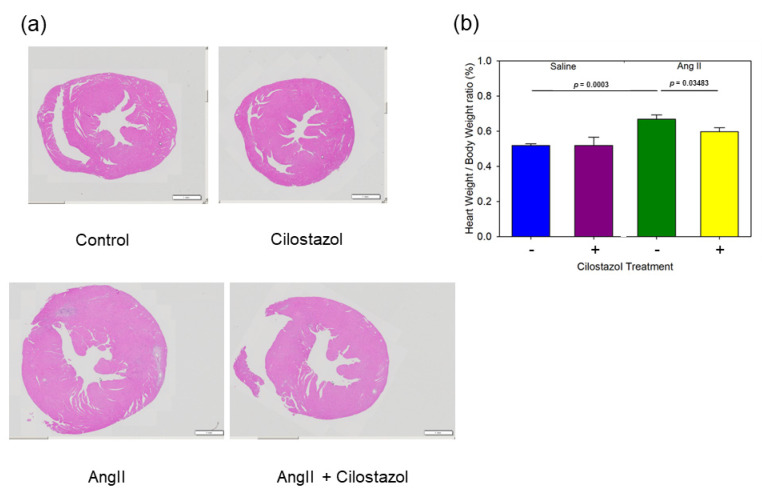
The effect of cilostazol on AngII-induced cardiac hypertrophy. (**a**) Representative images of transverse sections through the papillary muscle, stained with Hematoxylin and Eosin, are shown in each group. Control: saline/vehicle, cilostazol: saline/cilostazol, AngII: AngII/vehicle, AngII + cilostazol: AngII/cilostazol, the scale bar indicates 1 mm. (**b**) Mean heart weight/body weight ratio (%) of saline/vehicle (n = 6), saline/cilostazol (n = 5), AngII/vehicle (n = 11) and AngII/cilostazol (n = 9). Values represent the means ± SEM.

To evaluate the hypertrophic remodeling in response to AngII infusion, heart sections were stained with Hematoxylin–Eosin. Representative images of each group are shown (Figure 1a). AngII induced hypertrophy of heart, but cilostazol suppressed AngII-induced hypertrophy. We evaluated heart weight (HW)/BW ratio, a sensitive indicator of cardiac hypertrophy in mice. AngII increased the HW/BW ratio, while cilostazol significantly attenuated its increase by AngII (Figure 1b).

### 2.3. Cilostazol Suppressed AngII-Induced Cardiac Fibrosis

**Figure 2:**
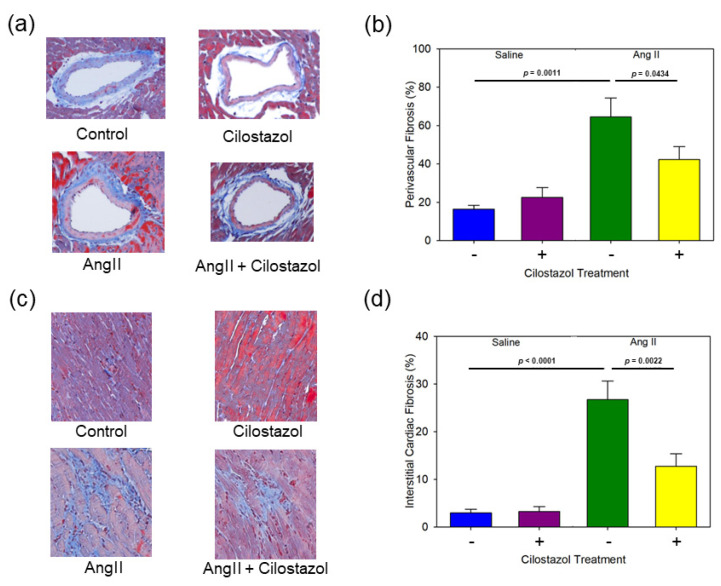
The effect of cilostazol on AngII-induced cardiac fibrosis. (**a**) Representative perivascular images of transverse sections stained with Masson’s trichrome are shown in each group. Control: saline/vehicle, cilostazol: saline/cilostazol, AngII: AngII/vehicle, AngII + cilostazol: AngII/cilostazol, magnification; 100×. (**b**) Mean perivascular fibrosis (%) of saline/vehicle (n = 6), saline/cilostazol (n = 5), AngII/vehicle (n = 11) and AngII/cilostazol (n = 9). (**c**) Representative images of fibrosis of myocardium of transverse sections stained with Masson’s trichrome are shown in each group. Control: saline/vehicle, cilostazol: saline/cilostazol, AngII: AngII/vehicle, AngII + cilostazol: AngII/cilostazol, magnification; 100×. (**d**) Mean fibrosis of myocardium (%) of saline/vehicle (n = 6), saline/cilostazol (n = 5), AngII/vehicle (n = 11) and AngII/cilostazol (n = 9). Values represent the means ± SEM.

To evaluate the cardiac fibrosis as remodeling in response to AngII infusion, heart sections were stained with Masson’s trichrome. The representative perivascular images of each group are shown in Figure 2a. AngII increased perivascular fibrosis but was significantly suppressed by cilostazol treatment (Figure 2b). Representative interstitial images of each group are shown in Figure 2c. Similarly, AngII increased interstitial fibrosis, but was significantly attenuated by cilostazol (Figure 2d).

### 2.4. The Effect of Cilostazol on mRNA Expression in Heart Tissue

**Figure 3:**
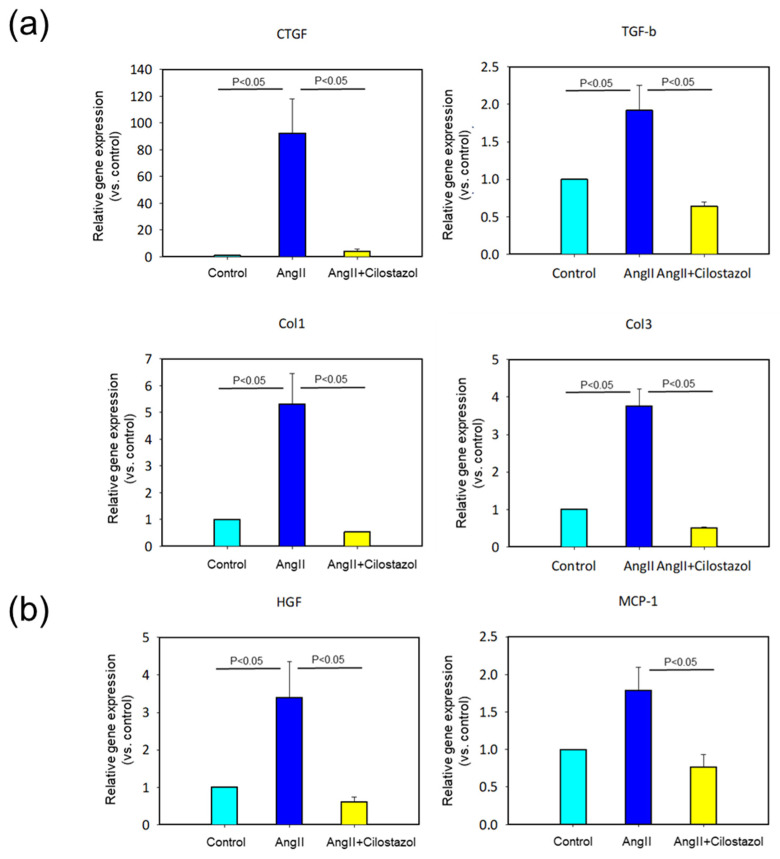
The effect of cilostazol on mRNA expression in heart tissue. (**a**) mRNA expression of fibrotic gene expression, CTGF, TGF-β, Col1 and Col3, in heart tissue were determined by qPCR (n = 6 in each group). Each bar represents the mean ± SEM of six experiments. (**b**) mRNA expression of inflammatory gene expression, MCP-1 and HGF, in heart tissue were determined by qPCR (n = 6 in each group). Each bar represents the mean ± SEM of six experiments.

To confirm the effect of cilostazol on myocardial fibrosis at the genetic level, we determined mRNA abundant of fibrous genes such as connective tissue growth factor (CTGF), transforming growth factor-β (TGF-β), collagen I and collagen III (Figure 3a). As expected, cilostazol significantly suppressed AngII-induced increases in expression of these fibrotic genes. Since it was reported that pro-inflammatory signals may promote fibrosis, next we determined mRNA abundant of inflammatory genes such as hepatocyte growth factor (HGF) and monocyte chemotactic protein 1 (MCP-1) (Figure 3b). As well as the fibrotic genes, cilostazol significantly attenuated AngII-induced increases in inflammatory gene expression.

### 2.5. The Effect of Cilostazol on Osteopontin Expression in Heart Tissue

**Figure 4:**
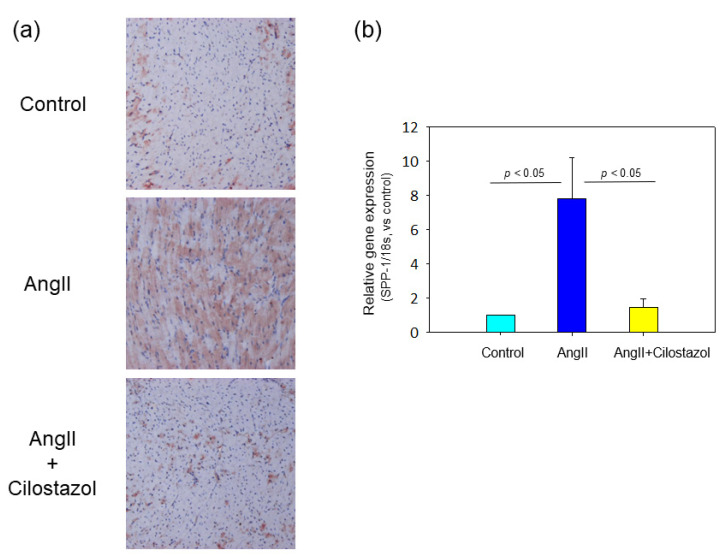
The effect of cilostazol on osteopontin expression in heart tissue. (**a**) Osteopontin expressions were evaluated in heart tissue by immunoperoxidase staining of osteopontin antibody. Magnification: 50×. (**b**) mRNA osteopontin gene expressions in heart tissue was determined by qPCR (n = 6 in each group). Each bar represents the mean ± SEM of six experiments.

OPN is known as an adhesion protein implicated as one important mediator of the profibrotic effects of AngII in heart [11,24,25]. Immunoperoxidase staining was performed with OPN antibody to evaluate the OPN protein expression of heart tissue. Representative images of each group are shown in Figure 4a. Cilostazol attenuated AngII-induced increases in OPN. Moreover, we evaluated mRNA abundant in OPN in heart tissue. Cilostazol significantly suppressed AngII-induced increases in OPN gene expression (Figure 4b).

### 2.6. The Effect of Cilostazol on AngII-Induced OPN mRNA Expression in Human Cardiac Myocyte In Vitro Study

**Figure 5:**
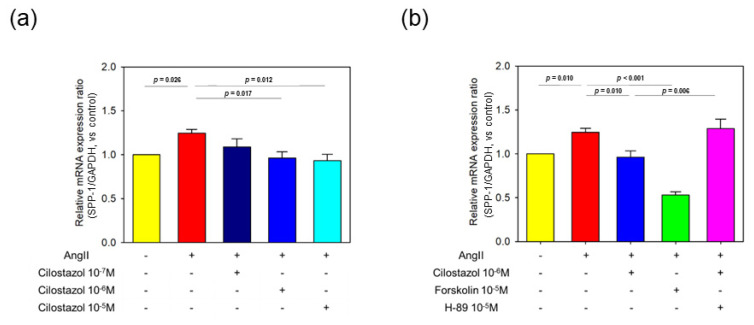
The effect of cilostazol on AngII-induced osteopontin mRNA expression in human cardiac myocyte; in vitro study. (**a**) mRNA expression of osteopontin was determined in AngII (0.1 μM)-stimulated human cardiac myocytes in the selected concentrations of cilostazol (0, 0.1, 1.0 and 10 μM), by qPCR (n = 6 in each group). Each bar represents the mean ± SEM of six experiments. (**b**) mRNA expression of osteopontin was determined in AngII (0.1 μM)-stimulated human cardiac myocytes in the presence or absence of cilostazol (10 μM)/forskolin (10 μM)/H-89 (10 μM), by qPCR (n = 6 in each group). Each bar represents the mean ± SEM of six experiments.

To further investigate the effects of cilostazol on cardiac fibrosis, we performed in vitro studies using Human Cardiac Myocytes (HCMs). HCMs were incubated with selected concentrations of cilostazol (0, 0.1, 1.0 and 10 μM) for 30 min and then incubated with AngII (0.1 μM) for 24 h. mRNA was extracted for real-time PCR. OPN mRNA expression was enhanced by AngII. Cilostazol suppressed the increment of OPN mRNA expression in a concentration-dependent manner (Figure 5a). Cilostazol is well known to increase intracellular cAMP concentration [26]. Thus, we hypothesized that activation of the cAMP-PKA pathway by cilostazol might be involved in the suppression of cardiac fibrosis. To elucidate the mechanism, we performed in vitro studies using forskolin, an activator of adenylate cyclase, and H-89, an inhibitor of PKA. HCMs were incubated with/without AngII (0.1 μM) and/or forskolin (10 μM) and/or H-89 (10 μM) and/or cilostazol (10 μM) for 24 h. Forskolin significantly attenuated AngII-induced enhancement of OPN mRNA level as well as cilostazol. H-89 canceled the effect of cilostazol on AngII-induced increases in OPN mRNA levels (Figure 5b). These results suggested that cilostazol attenuated AngII-induced OPN expression, in part, through a cAMP-PKA-dependent pathway.

## 3. Discussion

This study aimed to investigate the effect of cilostazol on cardiac fibrosis and analyzed the mechanism by which it acts to attenuate cardiac fibrosis elicited in mice with AngII. We found that cilostazol attenuated AngII-induced cardiac hypertrophy, perivascular fibrosis, and interstitial fibrosis, in association with the decrease in AngII-induced enhancement of fibrotic and inflammatory gene expression. In addition, we found that cilostazol attenuated OPN expression increased by AngII. Furthermore, we revealed that H-89, a PKA inhibitor, canceled the preferable effect of cilostazol on OPN expression induced with AngII. Thus, our study demonstrated that cilostazol attenuated AngII-induced increment in OPN expression, in part, through a cAMP-PKA-dependent pathway, resulting in a reduction in cardiac fibrosis.

Since HF is a leading cause of death, it is a major public health concern. Mortality rate within 5 years of diagnosis of HF is about 50% [1]. As the inadequacy of modern therapy is reflected in high mortality, a paradigm of new mechanisms for treatment is required. It commonly affects the older population, and given increasing life expectancy coupled with improved management of chronic medical conditions, the number of patients with heart failure is expected to increase. Thus, HF is a leading cause of mortality, morbidity, and poor quality of life. Cardiomyocytes, fibroblasts, and vascular cells in the heart are connected by a complex matrix principally composed of fibrillar collagen, which is instrumental in preserving structural integrity and plasticity. In the diseased heart, the matrix undergoes structural and subcellular changes that progressively influence heart function [27]. Accordingly, a major cause of cardiac dysfunction is deleterious tissue remodeling with interstitial fibrosis [5,28,29,30].

AngII induces severe perivascular and cardiac interstitial fibrosis in mice [10]. AngII exposure leads to significant ECM deposition that is characteristic of myocardial fibrosis [31]. It has been reported that this ECM deposition is correlated with an increase in key pro-fibrotic factors, TGF-β1 and its downstream mediator CTGF, shortly after AngII infusion. Administration of cilostazol in this study significantly improved increments of key pro-fibrotic factors. Moreover, evidence is provided that early cellular infiltration into the myocardium precedes significant ECM deposition, potentially providing pro-fibrotic stimuli to initiate fibrosis [31]. In this study, treatment of cilostazol attenuated inflammation factors (HGF and MCP-1), as well as key pro-fibrotic factors. These results suggest that cilostazol exerts anti-fibrotic and anti-inflammatory effects to attenuate cardiac fibrosis.

Cilostazol is a selective inhibitor of PDE3, which increases intracellular cAMP and activates PKA, thereby inhibiting platelet aggregation and inducing peripheral vasodilation [14]. Previous studies have reported that the cAMP pathway plays a key role in the effect of cilostazol in other tissues and cells including, bone, aortic endothelial cells and progenitor cells [32,33,34]. Cilostazol effectively activates the cAMP/Epac1 signaling pathway in liver tissue, thus attenuating liver fibrosis [22]. Multiple studies have previously established the association between cAMP and TGF-β1/CTGF [35,36,37]. In this study, treatment of cilostazol attenuated cardiac fibrosis by decreasing fibrotic factors and inflammation factors. These results complement those reports.

OPN has been implicated as a key factor in the development of interstitial fibrosis [24,38]. Moreover, OPN has been identified as a strong independent predictor of mortality in patients with chronic heart failure [39]. Thus, a proper regulation of OPN appears important for cardiac disease. AngII exerts a pro-fibrotic effect via induction of OPN. Our in vivo experiment revealed that AngII-induced OPN expression in the heart was suppressed by cilostazol treatment in a dose-dependent manner. In this respect, this is the first study to verify the effect of cilostazol on regulation of OPN expression as an indicator of fibrosis. Cilostazol suppressed cardiac fibrosis, as well as renal and liver fibrosis [21,22,23,40]. Forskolin, as a cAMP activator, suppressed AngII-induced OPN gene increased as well as cilostazol. However, this effect was reduced in the presence of H-89, a PKA inhibitor. It has been reported that OPN substantially impedes cAMP dependent anti-fibrotic signaling in cardiac cells [41]. These results suggest that cilostazol suppresses AngII-induced OPN expression, in part, through a cAMP-PKA dependent pathway, attenuating cardiac fibrosis. However, the exact mechanism by which OPN inhibits cAMP in cardiac myocytes remains to be elucidated in future studies.

Myocardial fibrosis is associated with the onset and progression of chronic HF in patients with hypertensive heart disease [42]. OPN is known to be a mediator that promotes fibrosis in fibroblasts [43,44]. The expression of OPN has also been correlated with the development of HF in both animal models [45] and clinical studies [46,47]. In fact, elevated OPN expression has also been shown to be related with the development of HF [48]. The beneficial effects of treatment with ACEi, ARBs and ARNI in patients with HF has already been documented, particularly in patients with systolic disease. On the other hand, the progression of cardiac diastolic dysfunction/diastolic ventricular failure with aging causes HF. Moreover, it has been reported that OPN is involved in senescence and fibrosis in the heart [49]. Thus, cilostazol, a PDE3 inhibitor, may prevent the onset of HF by suppressing OPN-induced cardiac fibrosis for any mechanism. Since cilostazol is already widely used in clinical practice, our results will provide a great promise in protecting against the cardiac fibrosis. The therapeutic effect of cilostazol on cardiac fibrosis in humans requires further study.

## 4. Materials and Methods

### 4.1. Mice and Study Protocol

Male, 8- to 12-week-old apoE^−/−^ mice were purchased from The Jackson Laboratory (Bar Harbor, Cat. No. 2052). All mice were maintained in a barrier facility, and ambient temperature ranged from 20 °C to 24 °C. Mice were fed diet and water ad libitum. Cilostazol was a generous gift from Otsuka Pharmaceutical Company. Either vehicle- or cilostazol-containing (0.1% wt/wt) diets were started 1 week prior to AngII infusion. Saline or AngII (1000 ng kg^−1^ min^−1^, Bachem, Cat. No. H-1705) was infused via Alzet mini-osmotic pumps (Model 2004; Durect Corp) for 28 days. Mini-osmic pumps were implanted subcutaneously on the right flank, as described previously [20]. The experimental protocol was approved by the Ethics Review Committees for Animal Experimentation of Okayama University Graduate School of Medicine, Dentistry, and Pharmaceutical Sciences (OKU-2012613).

### 4.2. Blood Pressure Measurement

Systolic blood pressure and pulse rate were measured by sphygmomanometry using a tail cuff system (BP-98A, Softron) following a published protocol [50]. Conscious mice were introduced into a small holder mounted on a thermostatically controlled warming plate and maintained at 37 °C during measurement.

### 4.3. Lipid Measurements

After an overnight fast, blood was obtained by cardiac puncture under anesthesia by isoflurane (1.5–3.0%, flow rate of 200–300 mL min^−1^, inhalation). Total cholesterol, triglycerides, and high-density lipoprotein cholesterol concentrations were determined in each plasma sample using commercially available enzymatic-based kits (Wako Chemicals Cat. No. 439–17501, No. 465–56701, and No. 432–40201).

### 4.4. Histological Analysis

The left ventricular wall, including papillary muscles, was fixed in 10% phosphate-buffered formalin and embedded in paraffin. Sections (10 μm) were stained with Hematoxylin–Eosin and Masson’s trichrome. Perivascular and myocardial fibrosis was evaluated in a blinded manner by two independent observers. Immunoperoxidase staining was performed to examine expression of OPN using an OPN antibody (Polyclonal Goat IgG, R&D Systems, Cat. No. AF808, AB_2194992) at 15 ug mL^−1^. Reactivity of the antibodies with tissue antigens was detected using AEC and ImmPACT AEC HRP Substrate (Vector Laboratories) as described previously [51].

### 4.5. Real-Time Polymerase Chain Reaction

mRNAs were extracted from ventricular tissues or cell lysates using RNeasy Mini kits (Qiagen, Cat. No. 74104, Hilden, Germany). Reverse transcription was performed using an iScript cDNA synthesis kit (Bio Rad, Cat. No. 1708891, Hercules, CA, USA). PCR reactions were performed with an ABI Step One Real-Time PCR System (Applied Biosystems, Quant Studio3, Foster, CA, USA) using Fast SYBR Green Real-time PCR Mixture (Applied Biosystems, Cat. No. 4385612, Foster, CA, USA) [52]. Primers for CTGF (Takara Bio Inc. Cat. No. MA028643), TGF-β (Takara Bio Inc. Cat. No. MA122075), collagen I (Takara Bio Inc. Cat. No. MA128559), collagen III (Takara Bio Inc. Cat. No. MA126692), OPN (Takara Bio Inc. Cat. No. MA114392) (Takara Bio Inc. Cat. No. HA201672), MCP-1 (Takara Bio Inc. Cat. No. MA066003), HGF (Takara Bio Inc. Cat. No. MA105799), and 18S ribosomal RNA (18S) (Takara Bio Inc. Cat. No. MA050364) and Glyceraldehyde-3-phosphate dehydrogenase (GAPDH) (Takara Bio Inc. Cat. No. HA067812) are available commercially. The mouse heart samples were normalized to values for 18s mRNA expression (ΔΔCT method). The human cardiomyocyte culture samples were normalized to values for GAPDH mRNA expression (ΔΔCT method).

### 4.6. Cell Culture and Treatment

HCMs were purchased from Promo Cell (Promo Cell, Cat. No. C-12810, lot. 2120301.2) and were grown in myocyte growth medium (Myocyte Growth Medium Kit: Promo Cell, Cat. No.C-22170). After reaching 80% confluence, HCMs were spread into 12 well plates. Each well had 1.5 × 10^5^ cells per well. After overnight serum starvation, HCMs were incubated with selected concentrations of cilostazol (0, 0.1, 1.0 and 10 μM) for 30 min. Then, HCMs are incubated with/without AngII (0.1 μM) and/or forskolin (Cell Signaling, Cat. No. 9803) (10 μM) and/or H-89 (Cayman chemical, Cat. No. 130964-39-5) (10 μM) and/or cilostazol (1.0 μM) for 24 h. mRNA was collected from each well. All experiments were performed in passages of 6 to 8.

### 4.7. Statistics

All plot and bar graphs were created with SigmaPlot v14.0 (Systat Software Inc.). All statistical analyses were performed using SigmaStat v3.5, incorporated into SigmaPlot v14.0. Data are presented as mean ± standard deviation or standard error of the mean, as appropriate. Statistical significance between multiple groups was assessed by two-way analysis of variance with Holm–Sidak post hoc one-way analysis of variance with Student–Newman–Keuls post hoc or one-way analysis of variance on Ranks with a Dunn’s post hoc, as appropriate. Values of *p* < 0.05 were considered statistically significant.

## 5. Conclusions

Cilostazol attenuates AngII-induced cardiac fibrosis in male apoE deficiency mice through activation of the cAMP–PKA pathway. Cilostazol may be a potential new therapeutic agent in cardiac disease.

## Figures and Tables

**Table 1:** Characteristics of the study mice.

	Control	Cilostazol	AngII	AngII + Cilostazol
n	6	5	11	9
BW (g)	26.9 ± 1.0	26.1 ± 1.0	26.2 ± 0.6	26.8 ± 0.7
SBP (mmHg)	97 ± 3	97 ± 3	127 ± 8 *	132 ± 7 **
HR (bpm)	621 ± 34	675 ± 23	645 ± 17	611 ± 15
T-Cho (mg/dL)	591 ± 48	584 ± 52	520 ± 109	524 ± 124
TG (mg/dL)	475 ± 190	391 ± 94	338 ± 78	379 ± 103
HDL-C (mg/dL)	22.0 ± 9.6	17.1 ± 6.5	17.4 ± 7.3	17.3 ± 4.9
LDL-C (mg/dL)	467 ± 31	484 ± 81	433 ± 97	426 ± 106
LDL/HDL	26.6 ± 17.3	32.7 ± 15.4	34.4 ± 28.0	25.6 ± 6.5

n: number, BW: body weight, SBP: systolic blood pressure, HR: heart rate, T-Cho: total cholesterol, TG: triglyceride, HDL-C: high-density lipoprotein cholesterol, LDL-C: low-density lipoprotein cholesterol, mean ± S.D., * *p* < 0.05 vs control (one-way ANOVA on Rank), ** *p* < 0.05 vs Cilostazol (one-way ANOVA on Rank).

## Data Availability

Not applicable.
